# Establishment of a reproducible and minimally invasive ischemic stroke model in swine

**DOI:** 10.1172/jci.insight.163398

**Published:** 2023-04-24

**Authors:** Carlos Castaño, Marc Melià-Sorolla, Alexia García-Serran, Núria DeGregorio-Rocasolano, Maria Rosa García-Sort, María Hernandez-Pérez, Adrián Valls-Carbó, Osvaldo Pino, Jordi Grífols, Alba Iruela-Sánchez, Alicia Palomar-García, Josep Puig, Octavi Martí-Sistac, Antoni Dávalos, Teresa Gasull

**Affiliations:** 1 Interventional Neuroradiology Unit, Department of Neurosciences, Hospital Universitari Germans Trias i Pujol, Badalona, Barcelona, Spain.; 2 Cellular and Molecular Neurobiology Research Group, Department of Neurosciences, Germans Trias i Pujol Research Institute (IGTP), Universitat Autònoma de Barcelona (UAB), Badalona, Barcelona, Spain.; 3 Department of Neurosciences, Hospital Universitari Germans Trias i Pujol, UAB, Badalona, Barcelona, Spain.; 4 Imaging Diagnostic Institute (IDI), Hospital Universitari Germans Trias i Pujol, Badalona, Barcelona, Spain.; 5 Center for Comparative Medicine and Bioimage (CMCiB) at IGTP, Badalona, Barcelona, Spain.; 6 Canon Medical Systems Spain and Portugal, Cornellà de Llobregat, Spain.; 7 Department of Cellular Biology, Physiology and Immunology, UAB, Bellaterra, Spain.

**Keywords:** Neuroscience, Stroke

## Abstract

The need for advances in the management/treatment options for ischemic stroke patients requires that upcoming preclinical research uses animals with more human-like brain characteristics. The porcine brain is considered appropriate, although the presence of the rete mirabile (RM) prevents direct catheterization of the intracranial arteries to produce focal cerebral ischemia. To develop a reproducible minimally invasive porcine stroke model, a guide catheter and guide wire were introduced through the femoral artery until reaching the left RM. Using the pressure cooker technique, Squid-12 embolization material was deposited to fill, overflow, and occlude the left RM, the left internal carotid artery, and left circle of Willis wing up to the origins of the middle cerebral arteries (MCAs), mimicking the occlusion produced in the filament model in rodents. Longitudinal multimodal cerebral MRI was conducted to assess the brain damage and cerebral blood supply. The technique we describe here occluded up to the origins of the MCAs in 7 of 8 swine, inducing early damage 90 minutes after occlusion that later evolved to a large cerebral infarction and producing no mortality during the intervention. This minimally invasive ischemic stroke model in swine produced reproducible infarcts and shows translational features common to human stroke.

## Graphical abstract

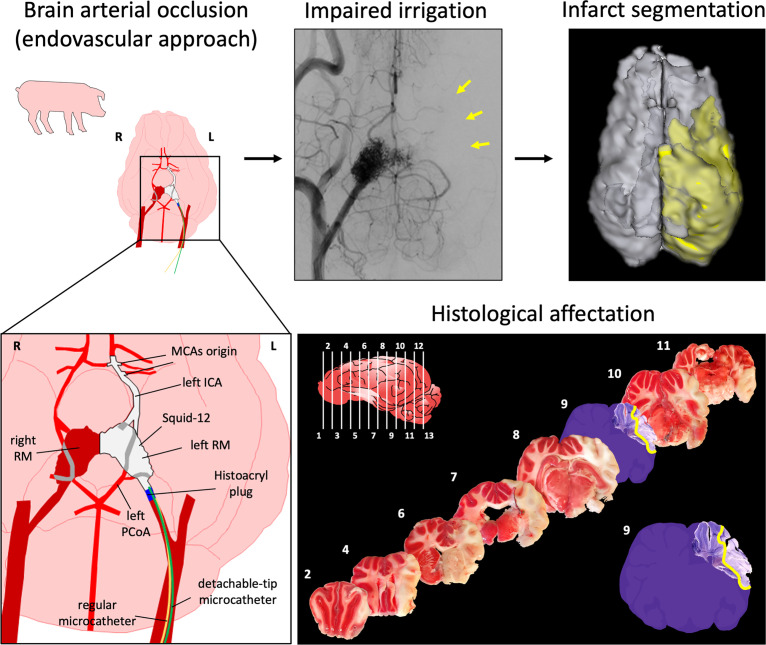

## Introduction

Endovascular thrombectomy revolutionized the management of acute ischemic stroke of large-vessel occlusion (1–6). Unfortunately, currently about 90% of patients are not eligible for endovascular thrombectomy, and after the procedure, almost 50% of patients do not have a good clinical outcome, despite successful revascularization (7, 8). For this reason, in addition to the improvement of recanalization techniques, it is imperative to find novel neuroprotective molecules or strategies useful in a long-lasting/permanent artery occlusion scenario (9) like that faced by most stroke patients worldwide because they are not eligible or do not have the opportunity to receive tissue plasminogen activator (tPA) or to benefit from thrombectomy (10, 11).

So far, most of the experimental therapies that have been tested preclinically in rodents with favorable results fail to deliver a positive result in the clinical arena. For a successful translation of the most promising experimental results from bench to bedside, Stroke Academic Industry Roundtable (STAIR) recommends that new therapies be tested in both permanent and transient experimental stroke models in large animals with human-like brain characteristics, using clinically relevant biochemical and MRI biomarkers (12–14). In this context, and unlike those of rodents, the pig brain resembles the human brain in terms of size, degree of gyrencephaly, or proportion of white/gray matter, and the use of swine in medical experimentation elicits minimal ethical and social concerns (15–17).

For years the modeling of ischemic stroke in swine has been achieved by exposing the middle cerebral artery (MCA) through craniectomy, followed by MCA occlusion by means of local ligation or electrocoagulation (18–21). Despite being rather invasive, these models have been useful for testing new treatments in permanent occlusion (22, 23).

The complex intracranial anatomy and the presence of the rete mirabile (RM) have so far hindered minimally invasive attempts to selectively occlude intracranial arteries through catheterization in swine (24). The RM is an intricate network of small vessels that impede the passage of catheters from the extra- to the intracranial vasculature, thus blocking access to the internal carotid artery (ICA), which runs exclusively intracranially in the pig. The occlusion of the ascending pharyngeal artery (APA) — the major vessel supplying the RM at the extracranial level — does not result in brain infarct due to the collateral blood flow through the contralateral APA and RM into the circle of Willis (CW). A previously attempted endovascular approach using embolic material produced only small infarcts in the basal ganglia (25). It was not until 2020 that Golubczyk et al. first reported a thrombin-induced ischemic stroke model in swine by endovascular access (26). The MRI-guided delivery of exogenous thrombin at the extracranial RM entry resulted in a thrombus that occluded the ICA and the MCA’s territories in 1 hemisphere. While allowing tPA-induced reperfusion, this model produced cortical ischemic infarcts that might have relatively high variability due to the difficulty in controlling the diffusion of thrombin in the blood (26, 27).

Here we describe a technical advance using an embolizing agent and the pressure cooker technique (PCT) (28) as a successful controllable endovascular method to produce reproducible, permanent, focal ischemic stroke in swine.

## Results

### Angiographic characterization of the arterial extra/intracranial interface in the pig.

**Figure 1:**
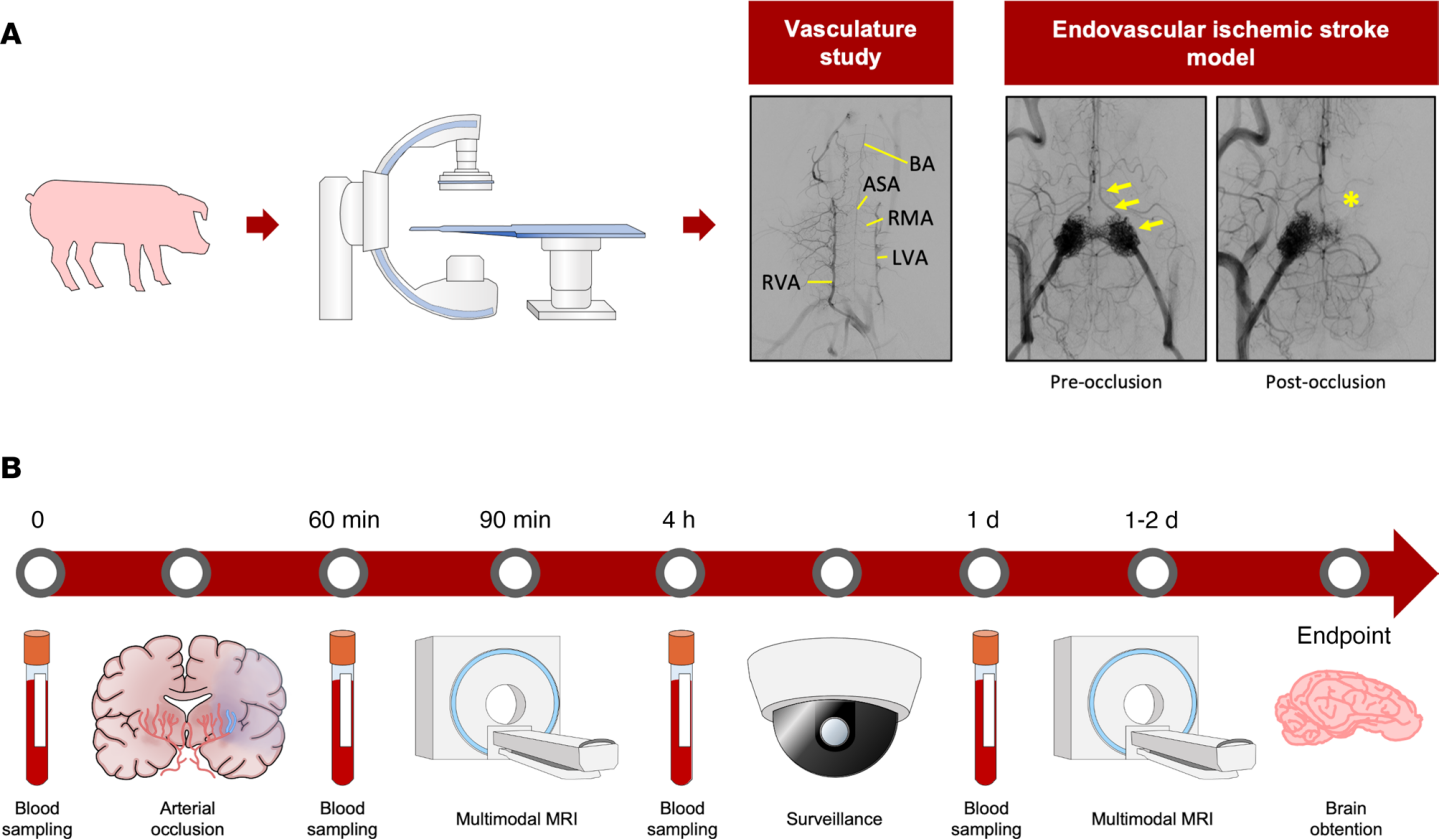
Experimental design and timeline infographic. (**A**) Digital subtraction angiography allowed the study of the vasculature (extracerebral-to-intracerebral arterial vessels). The vasculature study (*n* = 12) panel depicts a representative contrast image of the vertebral artery. The endovascular ischemic stroke model (*n* = 9) panel on the right depicts representative contrast images of the RM wings and distal circulation before (left image) and after (right image) embolization of the left RM and distal ICA. Yellow arrows in the left image indicate the contrast-filled RM and ICA/CW artery route that is no longer filled with contrast after embolization (yellow asterisk, right image). (**B**) Timeline of MRI acquisition and blood sampling. APA, anterior spinal artery; BA, basilar artery; LVA, left vertebral artery; RVA, right vertebral artery; RMA, radiculomedullary arteries.

In all the pigs, we used angiography to investigate any specific extra/intracranial arterial interface route able to provide direct endovascular access to the CW. In agreement with previous reports (29, 30), we found a complete CW in all of the pigs. Both carotids continued to the ascending pharyngeal arteries that gave rise to a butterfly wing–shaped, intricate, arterio-arterial plexus structure known as the RM, which has been previously reported to impede catheter navigation (24, 30). Each RM wing anastomosed into a single vessel, giving rise to a single ICA, which is part of the CW. In contrast to the human anatomy, the pig has no direct continuity of the vertebral arteries with the basilar artery. Instead, the communication between the vertebral arteries and the basilar artery is indirect, through small radiculomedullary arteries that contribute to the anterior spinal artery, which in turn continues with the basilar artery (Figure 1A, vasculature study). In our hands, none of the swine in the study had feasible access to the CW through navigating the left or right vertebral arteries with the catheters currently available for use in clinics for humans. Furthermore, despite minor interindividual peculiarities, the vascular architecture of the carotid/APA/RM/ICA showed a high degree of similarity in swine of different genetic background, age, and sex used in this study, supporting the generalization of the use of the model that we describe below.

### Training and exploratory procedures to design the ischemic stroke model.

The first 3 pigs in this study (1 Landrace × Pietrain, 1 Large White × Landrace, and 1 Specipig miniature pig) were used both for the angiographic study and to test the feasibility of a catheter to navigate from the APA into the RM. In these animals, the catheter was able to navigate the proximal third of the length of the RM, but it could not exit the RM to reach the ICA, instead rupturing the RM vessels, resulting in hemorrhage and death.

In the fourth pig, an embolization procedure was tested. Since the RM has been used as an experimental model for the treatment of arteriovenous malformations and/or for testing new embolization materials (28, 31, 32), we used the embolizing material Squid-12 propelled through an endovascular catheter into the left wing of the RM. This procedure resulted in a selective clogging of the left wing of the RM that prevented the blood supply to the brain through the left APA, and there was no impairment of blood flow through the right APA-RM wing. As a result, the contrast was unable to reach a small subcortical area of the ipsilateral brain hemisphere. In this pig, some of the flow to the left MCA territory was still available, probably due to the contribution of the anterior circulation. Accordingly, only a small subcortical infarct was measured in this animal, both in vivo in diffusion-weighted imaging–MRI (DWI-MRI) (4.2 cm^3^) and ex vivo using the tissue viability assessment in 2,3,5-triphenyltetrazolium chloride–stained (TTC-stained) brain slices. This animal was maintained sedated for the 3 days of experimental setup until it was euthanized.

### Development, characterization, and establishment of the stroke model.

**Figure 2:**
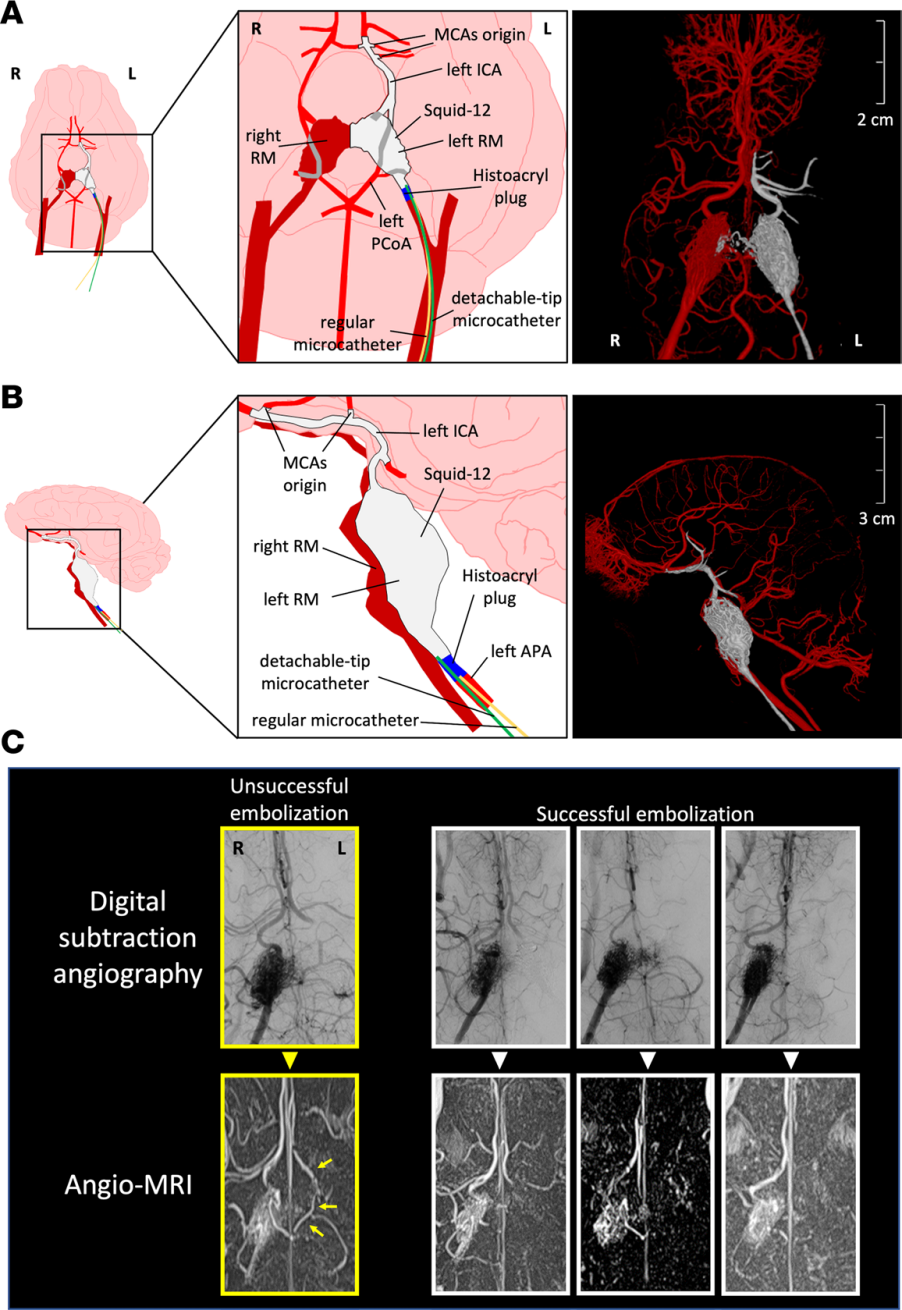
Schematic and in vivo arterial imaging of the occlusion generated in the model. (**A**) Schematic ventral drawing of the embolization procedure and setup and 3D anteroposterior projection. (**B**) Schematic sagittal drawing of the embolization procedure and setup and 3D sagittal projection. The artery areas embolized with LEA are depicted in gray, whereas arteries with functional lumen are depicted in red. Regular and tip-detachable microcatheters are depicted in yellow and green, respectively, and the Histoacryl plug, allowing the green tip to deliver the LEA while preventing reflux, is depicted in blue. (**C**) Representative digital subtraction angiography and angio-MRI images of the left RM + CW embolization of 4 pigs; pictures on the left show an unsuccessful embolization (framed in yellow; arrows indicate the patency of the left CW circulation). In the pictures of the other 3 pigs, the lack of contrast in the left RM and ICA/CW artery route is indicative of a successful embolization procedure (*n* = 7), hence meeting the standard of quality to be included in the study. APA, ascending pharyngeal artery (note that swine APA is equivalent to the extracranial portion of the ICA in humans); ICA, internal carotid artery; L, left; MCA, middle cerebral artery; PCoA, posterior communicating artery; R, right; RM, rete mirabile.

The results obtained from the training and exploratory procedures suggested that embolization of the left wing of the RM was not sufficient to obtain a large ischemic infarct. In order to obtain a more translational outcome, we designed a model of cerebral ischemic stroke using the liquid embolic agent (LEA) Squid-12 and PCT to embolize the left wing of the RM, left ICA, and left CW wing up to the origin of the MCAs (Figure 1A and Figure 2, A and B).

Seven Duroc × Landrace pigs (on average, 3.5 months and 41 kg) and 1 miniature pig from Specipig (17 months and 35 kg) were exposed to the Squid-12–induced permanent reduction of the blood flow in the left hemisphere. The procedure successfully occluded the CW/MCA connection in 7 of 8 pigs, as assessed by x-ray/digital subtraction angiography during the embolization procedure and confirmed by angio-MRI. Five of the 7 pigs with this successful occlusion survived at least 20 hours; 2 of them developed seizures, and the other 3 did not show external signs of serious seizures during the first 24 hours. In one of the pigs exposed to this procedure, Squid-12 failed to occlude enough CW length to affect the MCA origins/feeding points; this was a criterion of exclusion. The incomplete occlusion in this pig was also clearly observed in the 3D angio-MRI image at 90 minutes after the procedure (Figure 2C). No change in body temperature that could influence the stroke outcome was observed during the intervention.

### MRI follow-up study.

**Figure 3:**
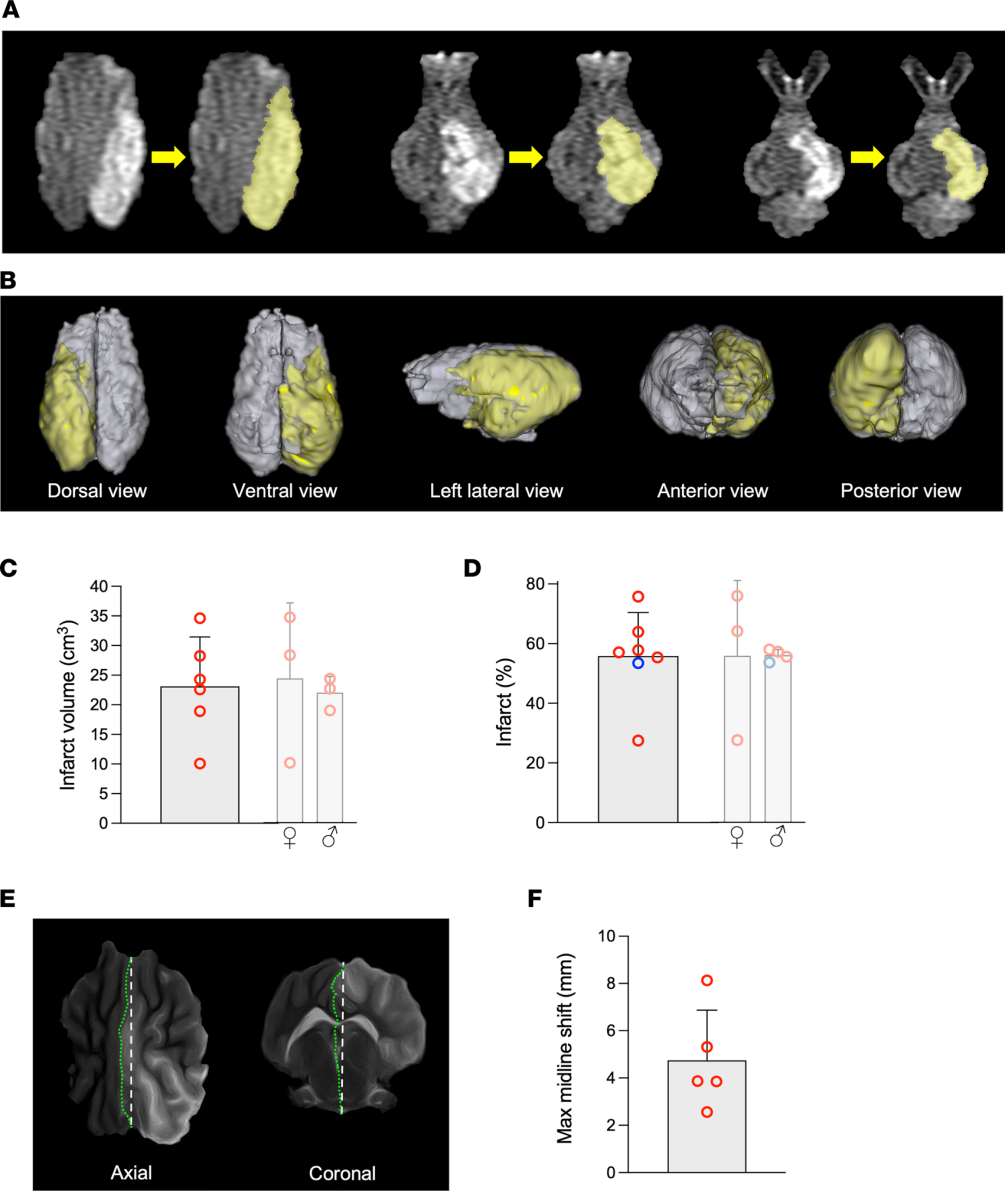
Infarct volume and midline shift determination through MRI. (**A** and **B**) 3D Slicer software was used to delineate and color (in yellow) the 2D hyperintense areas in DWI axial slice images to render a reconstructed 3D view of the damage in the whole brain. (**C** and **D**) Quantification of the maximal infarct for the 7 swine (6 Duroc × Landrace, plus 1 miniature pig (blue dot) in the infarct (%) graph) that underwent a successful occlusion of the CW/MCAs confluence, as expressed in absolute volume (**C**) and percentage of the ipsilateral hemisphere volume (**D**). Separate female and male graphs are also depicted (pale), showing a similar degree of infarction (female vs. male comparisons were made using the 2-tailed *t* test in **C** and the *U* test in **D**). (**E**) Axial and coronal MRI images showing the midline shift at 1 day (*n* = 5); straight broken line represents the theoretical midline, and the green line shows the actual midline. (**F**) Graph depicting the maximal midline shift at 1 day. Mean ± SD are shown.

**Figure 4:**
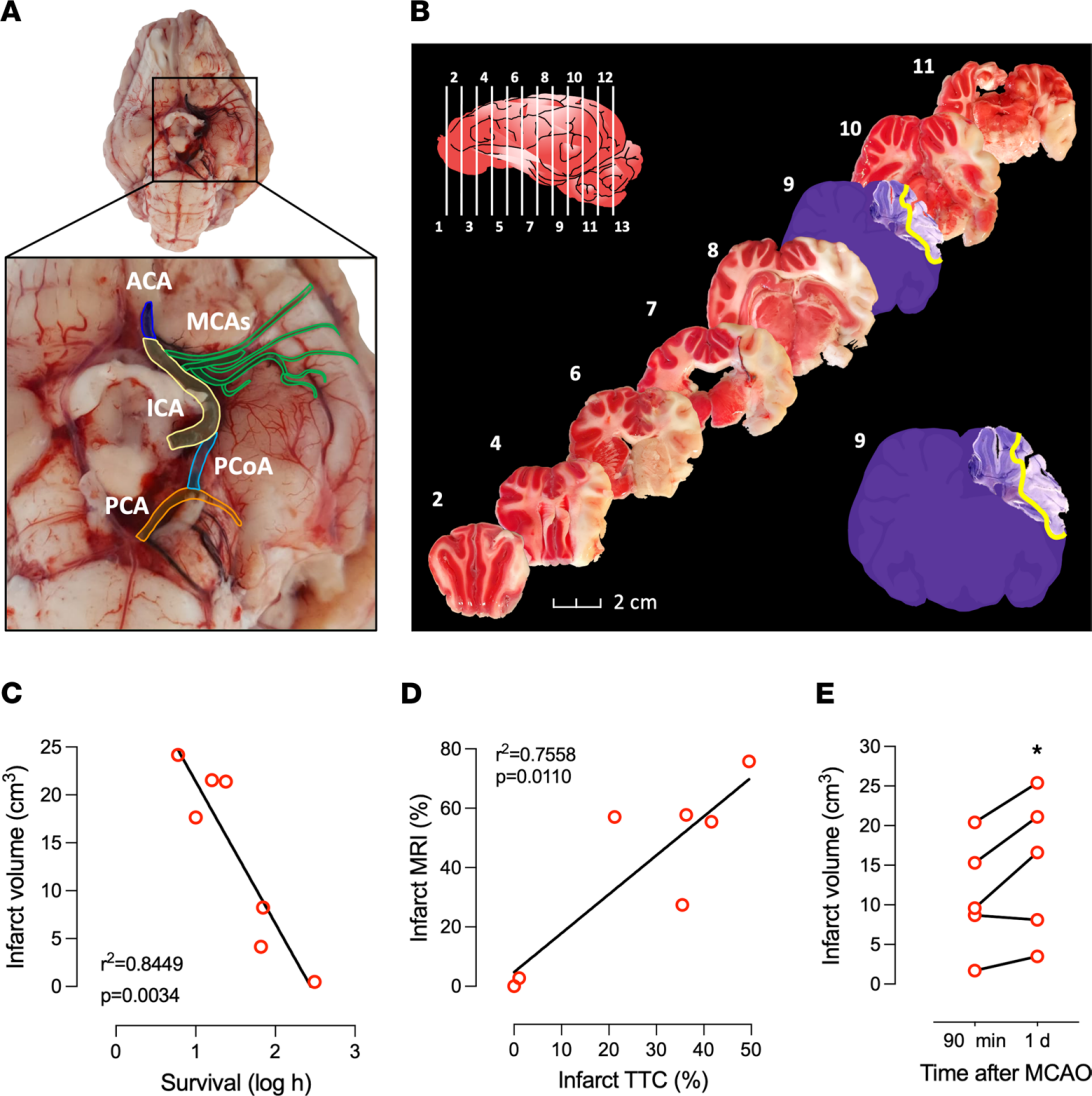
Ex vivo occlusion and brain infarct characterization, with infarct volume correlation to survival and infarct growth. (**A**) Ventral view of a swine brain, excised shortly after euthanasia, to assess the arterial filling with the black-colored LEA. The region of interest is zoomed in to show the main arteries in different colors. (**B**) TTC-stained coronal brain slices showing viable (in red) and infarcted (white) gray matter areas. The bottom right drawing shows the silhouette of cryopreserved section 9; slice fragment stained with cresyl violet shows the pale-stained infarct area (yellow line) that roughly matches the infarct in TTC sections 8 and 10. (**C** and **D**) Significant Pearson’s correlations (*n* = 7) between infarct volume and swine survival (**C**) and infarct volume (**D**) as measured by TTC or MRI. (**E**) Growth of infarct volume, corrected by edema, between 90 minutes and 1 day after MCAO (*n* = 5). **C** and **D** include data available for the pig with unsuccessful occlusion; these data are of interest for correlation purposes. MCAO, middle cerebral artery occlusion. ACA, anterior cerebral artery; ICA, internal carotid artery; MCA, middle cerebral artery; PCA, posterior cerebral artery; PCoA, posterior communicating artery. *P = 0.05 versus 90 minutes (paired *t* test).

**Figure 5:**
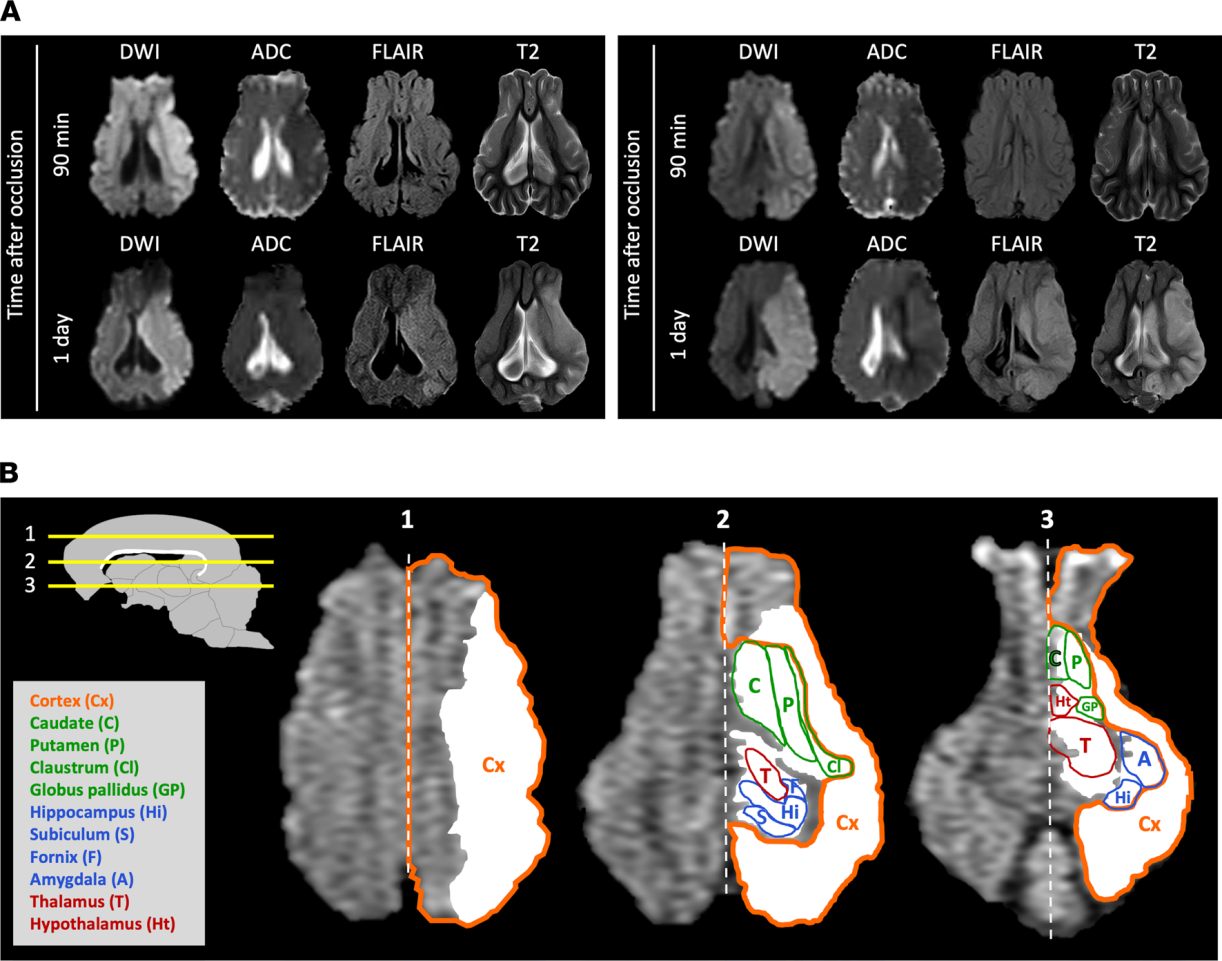
MRI differences between time points and brain regions affected. (**A**) Representative DWI, ADC, FLAIR, and T2 images of 2 different pigs at 90 minutes and 1 day after the embolization and ischemia onset. DWI and ADC abnormal signals (hyperintense and hypointense in DWI and ADC, respectively) are observed at 90 minutes in the ipsilateral hemisphere, whereas alterations in FLAIR and T2 are observed at 1 day. (**B**) Representative brain structures lesioned in the left CW embolization pig model. In white, an overlay of the area infarcted in 3 axial levels of T1 images (1, 2, and 3; upper left drawing). Superimposed on the white area, the colored contours of the cortex (orange), striatal (green), limbic (blue), or diencephalic (red) areas affected are shown.

**Figure 6:**
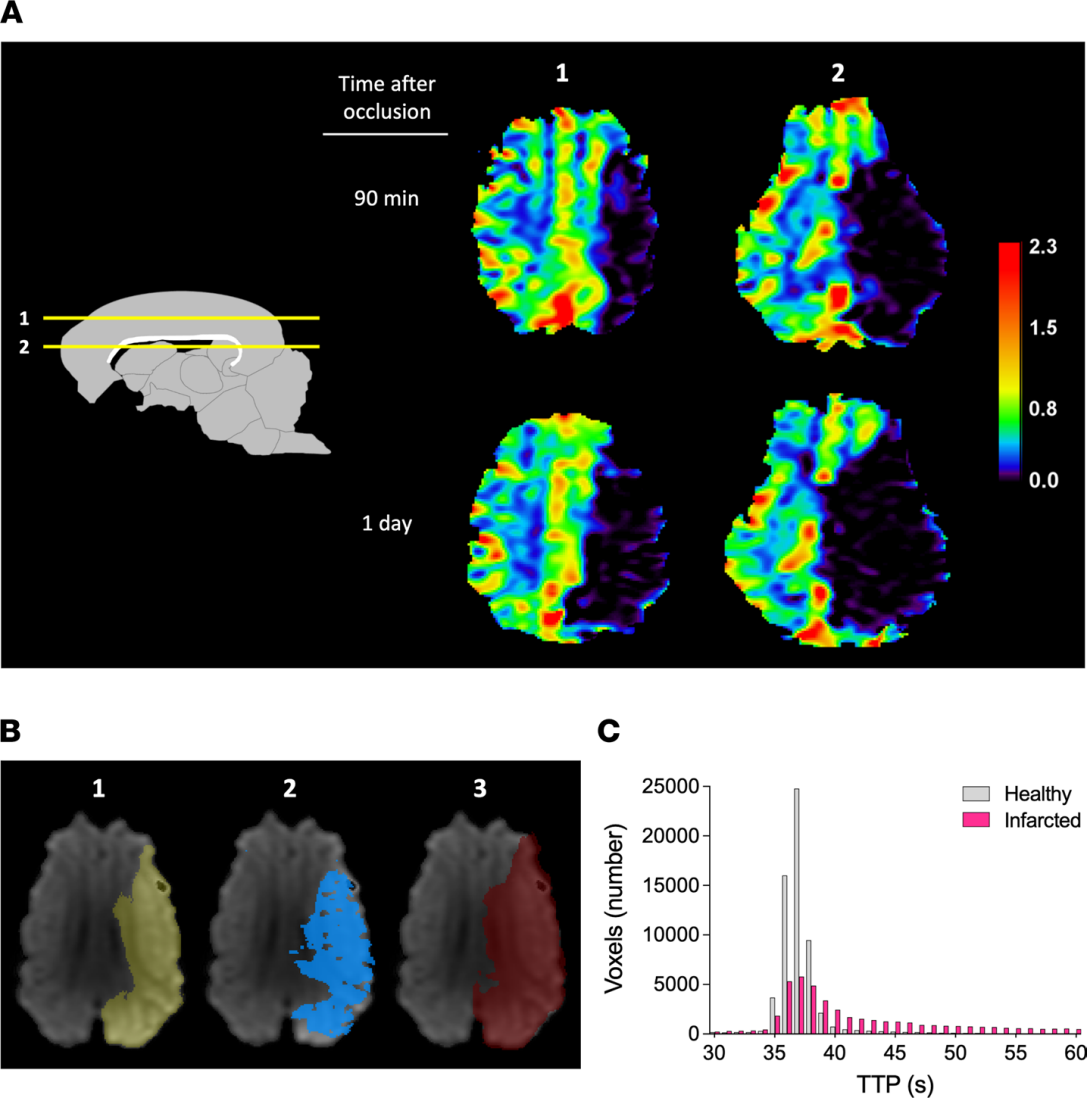
Brain perfusion impairment assessed through DSC-perfusion MRI. (**A**) Representative relative cerebral blood volume images 90 minutes and 1 day after stroke induction in 2 axial levels (1 and 2, as indicated in the drawing on the left). (**B**). Image 1 shows (in yellow) the area of brain infarction measured in DWI at 90 minutes; image 2 shows (in blue) a map of brain tissue with a time-to-peak (TTP) greater than 40 seconds obtained 90 minutes after the stroke onset; image 3 shows (in red) the maximum area of infarct measured 1 day after the ischemia onset. (**C**) Histogram showing the number of voxels as a function of TTP in infarcted tissue (fuchsia bars) and healthy specular contralateral ROI (gray bars) 90 minutes after occlusion. The TTP > 40 threshold was selected by comparing the number of voxels in both healthy and infarcted tissue for each given TTP time point. Most healthy tissue voxels show a TTP of 36–38 seconds, whereas voxels in the infarcted areas depict a TTP greater than 40 seconds; a TTP delay of 4 seconds might mark compromised tissue.

**Figure 7:**
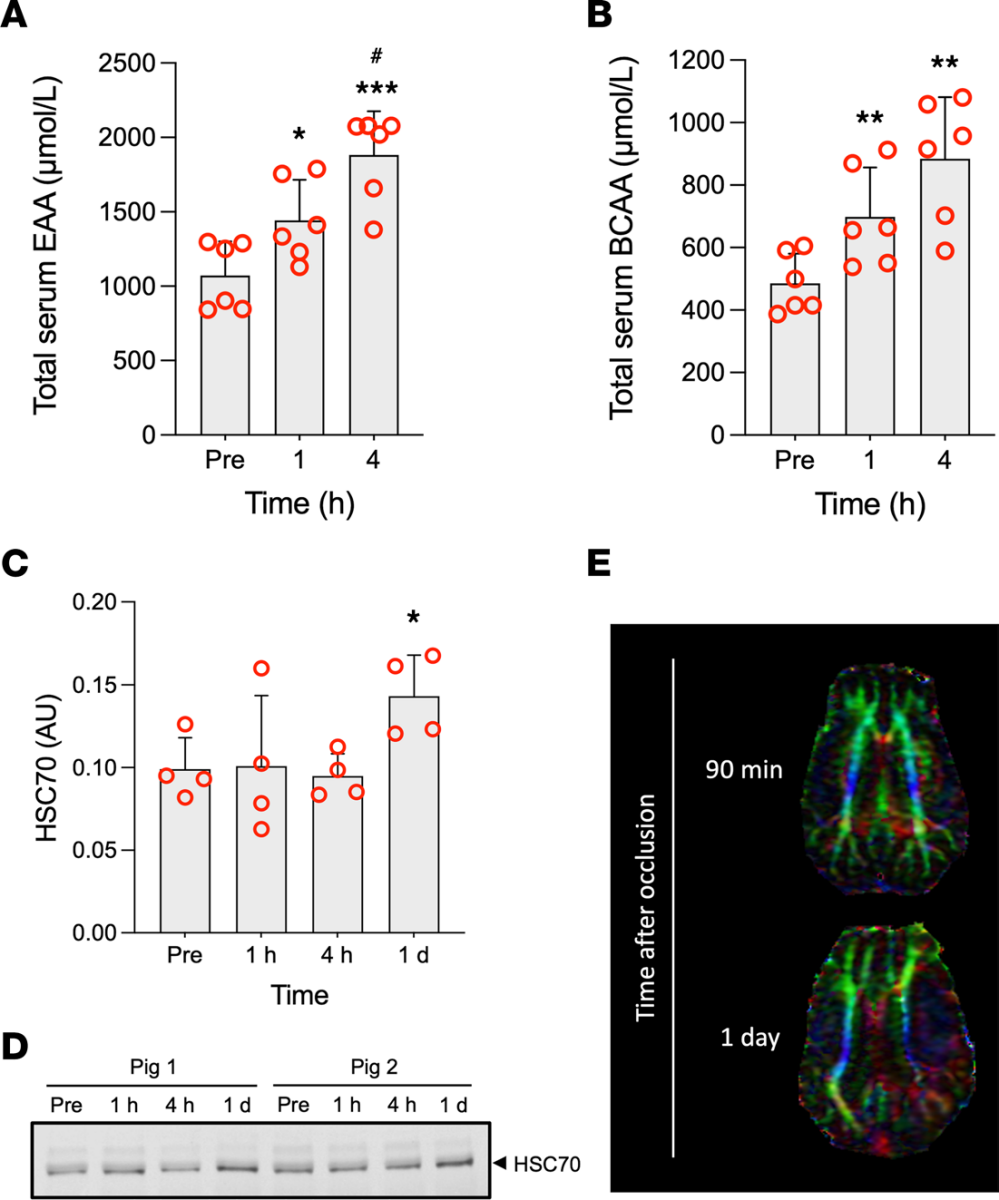
Time-course effect of stroke. (**A**–**C**) Time-course effect of stroke on the total serum levels of essential amino acids (EAA, *n* = 7), branched chain amino acids (BCAA, *n* = 7), and plasma HSC70 levels (*n* = 4, animals included are those with samples at all time points). (**D**) Representative Western blot image of plasma HSC70 levels of 2 pigs before (Pre) and 1 hour, 4 hours, and 1 day after the onset of stroke. (**E**) Diffusion tensor imaging (DTI) showing the white matter architectural complexity of the pig brain 90 minutes or 1 day after the stroke onset; color codes of the tract orientation are as follows: red for fibers crossing left-right, green for anterior-posterior, and blue for superior-inferior. At 90 minutes after stroke onset, the white matter tracts in the right and left hemisphere mirror each other. At 1 day after stroke onset, the left hemisphere superior-inferior tract structure shows damaged/thinner compared with the right contralateral hemisphere. *P < 0.05, **P < 0.01, ***P < 0.001 versus Pre, ^#^*P* < 0.05 versus 1 hour (1-way ANOVA with Tukey post hoc test). Mean ± SD are shown.

The mean ± SD infarct volume was 22.8 ± 8.3 cm^3^ 1 day after stroke onset; this area is more than 50% of the ipsilateral hemisphere (Figure 3D). All the pigs that successfully underwent the procedure to occlude the CW/MCA intersections showed some swelling of the ipsilateral hemisphere with a midline shift in T2-weighted imaging (Figure 3, E and F) and developed mixed cortical and subcortical infarcts as assessed in DWI at 1–2 days after occlusion or TTC staining (Figure 4B and Figure 5). The study of the early evolution of stroke damage in DWI b1000 and apparent diffusion coefficient (ADC) axial maps showed signs of early ischemic damage after only 90 minutes of complete occlusion (Figure 5A). The DWI-measured infarct corrected by edema grew 34% when comparing results 90 minutes after occlusion with those observed 1–2 days later (Figure 4E). Hypoperfused areas, as calculated based upon relative cerebral blood flow, were found to encompass the territory areas that ultimately developed infarct. The relative cerebral blood volume of hypoperfused brain tissue assessed 1 day after the ischemia was larger than that observed at 90 minutes after stroke onset (Figure 6A), indicating an expanding area of perfusion deficit. This observation fits with the time-course increase of hypoperfused brain areas observed in rats along the occlusion period in the filament thread model of ischemic stroke (33, 34). Ninety minutes after the occlusion, the time-to-peak (TTP) in the ipsilateral DWI-affected areas was ≥ 40 seconds, whereas the TTP in the healthy specular contralateral region of interest (ROI) was 36–38 seconds, suggesting that a TTP delay ≥ 4 seconds indicates compromised tissue (Figure 6, B and C). When available, diffusion tensor imaging (DTI) maps obtained after 1 day showed a displaced and thinner anteroposterior tract in the ipsilateral ischemic hemisphere (Figure 7E). Supplemental Figure 1 (supplemental material available online with this article; https://doi.org/10.1172/jci.insight.163398DS1) depicts representative images of the different multimodal MRI sequences obtained in the study. The DTI color-coded maps obtained at 90 minutes after stroke onset showed that the ipsilateral ischemic hemispheres nicely mirror those of the unaffected contralateral one, suggesting that white matter tracts are well preserved in the initial hours (Supplemental Figure 2). No signs of hemorrhage were observed in the T2* (Supplemental Figure 3). Both T2* and flow-sensitive black blood sequence maps revealed asymmetrical prominent hypointense signals in deep subcortical areas of the ipsilateral hemisphere that were not attributable to an LEA-derived artifact (35).

### Neurological dysfunction after stroke.

After stroke induction, the animals presented neurological symptoms such as ataxia, lameness, nystagmus, tremors, repetitive head shakes, circling toward the ipsilateral damaged hemisphere, head pressing to the walls, and occasional seizures of moderate or high intensity. The pigs’ neurobehavior was video recorded during the first night, and in addition, a 60-minute period was video recorded from 9–10 a.m. each day (a representative video of a pig’s behavior after stroke is included as Supplemental Video 1). Following seizure episodes longer than 30 seconds, and since the protocol of the study did not include antiepileptic treatment, the animals were then sedated with midazolam and euthanized as soon as possible.

### Postmortem brain infarct assessment in coronal brain slices.

Using the TTC method of staining fresh ex vivo brain slices, the ischemic lesion was observed in the cortex and subcortical areas/nuclei of the striatum, thalamus, and hippocampus. These areas are consistent with the damaged areas observed in the 3D reconstruction of MRI (3D slicer; Figure 3, A and B). Unstained regions of gray matter in the TTC-stained sections matched pale-colored Nissl areas, indicative of cellular loss in the brain parenchyma (Figure 4B).

### Ischemic stroke blood biomarkers assessment.

A time-course increase in the serum levels of essential amino acids, and also branched chain amino acids considered alone, was observed starting 1 hour after stroke onset as compared with prestroke levels (Figure 7, A and B). A specific increase was observed for L-phenylalanine and L-methionine (Supplemental Figure 4). One day after stroke onset, we found increased plasma levels of heat shock cognate 71 (HSC70) (Figure 7, C and D), a protein of membrane domains involved in the exosome release (36); HSC70 blood levels in the acute phase of stroke have predictive value for the poststroke development of late-onset seizures (37).

## Discussion

We here report a reproducible, minimally invasive, and permanent ischemic stroke model in swine fit to represent the largest proportion of stroke patients: those who are not eligible for tPA or thrombectomy treatment and those who are eligible but show futile recanalization. This model will help to bridge the translational gap between preclinical research and medical implementation. We established the model by clogging the left RM wing to the CW/MCA intersections using the embolization substance Squid-12 and the PCT; PCT is currently used in clinical practice to treat arterio-venous malformations and to embolize structures with difficult vascular access.

The embolization procedure with Squid-12 shows several similarities to the intraluminal filament thread model in rodents (38, 39), which is probably the most used technique in preclinical studies in rats and mice. Infarct of the MCA territory is achieved in the intraluminal thread rodent model when the thread completely fills and clogs the lumen of the ICA-CW up to the confluence with the MCAs for at least 60–90 minutes. The technique we present here in the pig clogs the same arteries by using a progressing embolization compound to circumvent the difficulty of the extreme tortuosity and small caliber of the RM vessels that, for the tools currently available, act as a natural barrier to the superselective catheterization of the ICA and distal CW to the origin of the MCAs. Furthermore, the present technique allows the direct, real-time monitoring of the embolus (Squid-12 is radiopaque) and the management of its progression and effects on the blood supply to the ROI of the ipsilateral brain hemisphere (see arrows and asterisk in Figure 1A). Our pig model resembles the large-vessel occlusion in human ischemic stroke that is known to be associated with a mortality of 26% at 90 days even with the best medical treatment (40). Mortality is also significant in our model, as a 28% mortality rate was observed in the overnight postinfarct onset, similar to the mortality in the filament thread model in rodents. In the swine model, a negative correlation was found between infarct volume and survival time (Figure 4C). Nonetheless, since we observed profuse internal bleeding in the femoral zone in 1 of the 2 pigs that died overnight, overnight survival might still be increased by the prevention of rebleeding in the femoral catheter access. The remaining pigs of the study were euthanized in the days following the stroke onset to prevent self-inflicted injuries associated with the seizures. At present, to further characterize the model, additional animals are being treated daily after stroke induction with the antiepileptic levetiracetam, thus preventing the animals from showing external signs of seizures for at least 1 week. The pigs survive at least 1 week, despite that they hold a quite large infarct lesion (Supplemental Figure 5 and Supplemental Videos 2 and 3) (no further time points are investigated).

Signs of ischemic damage (hyperintensity on DWI with a corresponding hypointensity on the ADC map) were observed as early as 90 minutes after occlusion. This early effect is in agreement with evidence of early ischemic damage in the brain parenchyma on DWI detected around 30 minutes after intraarterial thrombin administration reported in the model by Golubczyk et al. (26). In our model, the presence and anatomical location of this early ischemic damage were confirmed in subsequent DWI/ADC maps and, additionally, in hyperintense areas on fluid-attenuated inversion recovery (FLAIR) and T2 maps obtained at 1–2 days relative to the contralateral hemisphere (Figure 5A). All the pigs with complete occlusion of the left RM and the CW wing up to the MCA developed large ischemic infarcts involving 57% of the ipsilateral hemisphere, encompassing both cortical and subcortical regions (Figure 3A, Figure 4B, and Figure 5), and the infarcts showed midline shift after 1-day evolution (Figure 3, E and F) in accordance with reports in other models (41, 42). Moreover, infarcts of similar size were observed in male and female pigs in our model (Figure 3, C and D), with females showing more variability. Neither T2* sequences nor ex vivo assessment in brain slices showed any signs of hemorrhage in our model, in contrast to what has been reported in other invasive swine models (41, 43).

The 3D reconstructions of ischemic lesions obtained in axial MRI before euthanasia correlated with the infarct volume observed in the ex vivo TTC stain of fresh brain coronal slices (Figure 4D). The infarct volume mean ± SD obtained in our model was 22.8 ± 8.3 cm^3^, exceeding the average infarct previously reported in the model that approaches direct in situ transient or permanent occlusion of the MCAs through craniectomy and/or enucleation of an eyeball (41–44), the less invasive transient ischemia induced by endothelin (45, 46), or the minimally invasive thrombin model (26). Brain infarct was observed 1 day after occlusion in our model in claustrum and putamen, fornix and hippocampus, cortical areas of the visual and somatosensory cortex, temporal gyrus, and prepiriform and piriform cortex in 100% of the animals (Figure 5B and Supplemental Table 2); the major brain areas affected are the same as those previously reported for the permanent, invasive, and direct occlusive MCAs model (42). A certain volume of infarction was already observed in at least 70% of the pigs in the above-listed cortical areas 90 minutes after the occlusion, whereas the prefrontal, orbitofrontal, or anterior cingulate cortices showed damage in only 20%–33% of the animals 90 minutes after the occlusion. Ninety minutes after onset of permanent occlusion, the white matter tracts in the right and left hemispheres mirrored nicely. On day 1 after stroke onset, the ipsilateral superior-inferior tract structure was thinner than the contralateral tract, an effect that might result from edema associated with the model.

The Squid-12 was clearly observed filling the MCA lumen in some animals (Figure 4A), but embolization of the MCAs lumen is not mandatory to produce infarct of the MCA territory when the connection/pipeline between the CW and the MCAs is clogged. Squid-12 requires prolonged injections to precipitate and achieve a proximal plug. However, by making a proximal plug with Histoacryl in the PCT, we successfully prevented Squid-12 reflux and obtained an excellent LEA progression into the ICA reaching the distal MCA branch and even reaching the initial portion of the anterior cerebral artery. Ours is a model of permanent ischemic stroke, with the Squid-12 remaining at exactly the same location along the different angio-MRI time-course scans. A limitation of this approach is that it requires neurointerventional expertise to get a correct proximal plug, to achieve the distal progression of the Squid-12, to avoid the occlusion of the contralateral wing of the RM, and to precisely control the occlusion site.

The infarct grew moderately in this pig stroke model (a 34% increase when corrected by edema) in the interval between the first MRI-DWI acquisition at 90 minutes and 1 day after occlusion (Figure 4E), providing an area of tissue at risk within a suitable time window to test neuroprotective infarct progression even when blood flow cannot be restored. In fact, the area susceptible to being protected might be larger than that calculated since MRI-ADC/DWI lesion volume in the early hours after stroke onset has been reported that (a) overestimates the area of infarct compared with that of TTC staining in a swine model (46) and (b) includes areas susceptible to lesion reversal in human stroke (47). Our model stops blood flow from the left wing of the CW to the confluence of the MCAs, minimizing variability due to inadvertently leaving one of the MCAs perfused in the animals that have several left MCAs.

Reproducibility is clearly an asset for a translational model intended to be used in cerebroprotection preclinical studies to test new therapies. Several neuroprotective treatments have been reported to be successful in permanent occlusion models in rodents (33, 48) and recently in a permanent occlusion model in pigs (22), suggesting that there are opportunities for protection while in occlusion/ischemia. The recent publication of Porcine Neurological Imaging templates (49, 50) and the open-source 3D printable stereotaxic systems (51) mean that there is also a clear advantage in adopting pigs as the translational model of choice to test new therapies.

Our model has proved to mimic some features of human stroke: (a) the presence of blood biomarkers common to ischemic stroke models and human stroke, such as the increased blood levels of essential amino acids recently reviewed (52) (including a specific increase of phenylalanine or methionine; refs. 52, 53) or of HSC70 (blood HSC70 levels have been reported to be associated to the development of late-onset seizures in stroke patients; ref. 37); (b) the compromised white matter integrity 1 day after stroke, in agreement with other swine ischemic models (41, 42); and (c) the brain edema and midline shift in a preserved skull, potentially reproducing the high intracranial pressure conditions observed in patients with large infarct volumes. This reliability regarding equivalent effects of stroke in humans and our pig model ought to be viewed as an experimental asset of the model for discovery studies.

In conclusion, we have developed, characterized, and established a potentially novel, minimally invasive, and reproducible model of focal permanent ischemic stroke in swine. This model reproduces features of stroke in humans in a big mammal with significant brain physioanatomical resemblances to humans — e.g., a high degree of gyrencephaly, white/gray matter ratio, acute/hyperacute biomarkers in blood, and neuroimaging markers. The use of this model in an animal species with a brain with human-like characteristics, such as the specific gyrencephaly adapted vascular architecture or the specific gyrencephalic white matter architecture, will significantly influence the outcomes of studies addressed at unraveling the pathophysiology of thrombotic/ischemic processes.

## Methods

### Study design.

This study was designed to establish a potentially novel, reproducible, and minimally invasive model of ischemic stroke in swine. The sample size was determined by power analysis, considering the infarct volume at 1–2 days after stroke onset as the primary outcome. The criteria for inclusion were preestablished as a successful blockade of the blood flow through the left MCAs, and endpoints were prospectively selected by animal welfare criteria; these included the following as final humanitarian endpoints: any complication during intervention or at the initial postintervention with the anesthetized animal and clear signs of suffering and/or distress, not reversed by supervision protocols. To test the reproducibility of the model, power analysis calculation was manually performed by 2 independent expert statisticians who indicated that 6 animals are required to estimate the average infarct volume obtained with a precision of ± 20 percentage points assuming a SD of 25% in a 2-sided 95% CI. The final sample size was set to 8 animals to account for possible withdrawals during the experimental set up. The primary objective of the research was to obtain a reproducible endovascular ischemic stroke; therefore, no need for randomization or blinding was specifically required in this study. Nonetheless, infarct size calculations and biomarker assessment were performed blindly by investigators.

### Animals.

A total of 12 pigs (average weight 40.1 ± 3.3 kg, 50% female and 50% castrated male) with different genetic backgrounds were used in this study. Ten 4-month-old hybrid pigs (1 male Landrace × Pietrain, 1 female Landrace × Large White, 5 female Duroc × Landrace, and 3 male Duroc × Landrace) were obtained from Mir Ramadera; two 17-month-old male miniature pigs were obtained from Specipig (Specific Pig S.L.). Upon arrival at the CMCiB animals were housed undisturbed in their pens for a minimum of 1 week.

Significant differences in macroscopic brain anatomy have been recently reported between strains/breeds of swine (54), so we used pigs of different genetic backgrounds in an angiographic study to search for anatomical vascular differences that could provide feasible access of the catheters to the CW, either through the RM or the vertebral arteries. In compliance with the 3R strategy to “reduce, refine, and replace,” when possible, the same swine provided data for more than 1 of the following objectives: (a) the angiographic study, (b) the catheterization and crossing attempt through the RM, (c) the training of the occlusion/embolization of the left wing of the RM, and (d) the occlusion/embolization of the left wing of the RM + left ICA + left side of the CW.

### Preneurointerventional procedures.

All procedures required for the radiological study of the anatomy, the neurointerventional embolization, and MRI acquisition were performed under general anesthesia, mechanical ventilation, and sterile conditions.

Swine were deprived of food for 12 hours before anesthesia, and detailed hemograms were obtained to assess their physiological state. Complete blood cell counts and other blood biochemical parameters assessed included levels of blood urea nitrogen (BUN), creatinine (CRE), alanine aminotransferase (ALT), alkaline phosphatase (ALP), aspartate aminotransferase (AST), total bilirubin (TBIL), glucose (GLU), total protein (TP), albumin (ALB), globulins (GLOB), calcium (CA), and electrolytes. Hemoglobin and hematocrit, as well as erythrocyte, leukocyte, and platelet counts, were also determined.

Analgesic/sedative premedication (0.04 mg/kg atropine, 3 mg/kg ketamine, 0.03 mg/kg dexmedetomidine, 0.3 mg/kg midazolam, and 0.01 mg/kg buprenorphine) was administered i.m. Once sedated, the animals were given oxygen by face mask and were administered the antibiotic tulathromycin (2.5 mg/kg) i.m. Anesthesia was induced by a slow i.v. injection of propofol (1–2 mg/kg). Once deeply anesthetized, tracheal intubation was performed to allow the connection to a semi-closed anesthetic mechanical ventilation support system (fraction of inspired oxygen [FiO_2_] 45%–60%) with volume ventilatory control with maintenance anesthesia using isoflurane (1.5%–2.5%). Thereafter, the pigs were transferred to a dedicated large animal neurointerventionism suite to obtain in vivo vascular images using a Canon Alphenix monoplane angiography system (Canon Medical Systems Corporation) and a corneal protector (Lipolac, 2 mg/g) was applied. Vital signs (ECG, heart and respiratory rates, oxygen saturation, capnography, and arterial blood pressure) were continuously monitored throughout the procedure. During the preoperative and intraoperative phases of the intervention, body temperature was assessed by means of a rectal temperature probe, and a forced-air warmer (3M Bair Hugger 77514 240 V-SPA-A) was used to maintain the body temperature (Supplemental Table 3). Interventionism procedures were conducted with the pig in supine position, and MRI was conducted with the pigs in prone position.

To prevent thrombi formation, physiological saline containing 3,000 heparin units was administered in a boost during catheterization, and 1,000 additional heparin units were infused every 30 minutes during the intervention. Noradrenaline was infused 0.6–26 μg/kg/h when required to stabilize cardiac frequency.

Introducer 7F sheaths (Terumo Europe) were placed into both femoral arteries by puncture guided by ultrasounds. The angiographic study in the Alphenix system was performed using 4F pigtail, 4F vertebral catheters, and Terumo Glideware (Terumo Europe España) that were navigated into the right or left APA. Iodinated contrast agent Ioversol (Optiray, Mallinckrodt Medical Imaging Ireland) was injected through the catheters for guidance and angiographic imaging purposes. Projections, rotational studies, and 3D reconstructions were performed with Canon proprietary software to obtain the intracranial/extracranial vasculature map (Figure 1A).

### The x-ray–guided endovascular permanent obstruction of blood supply to the left MCAs using the PCT.

Attempts to reach the CW directly by catheterization were made in the first 3 pigs in the study, resulting in failure and/or an end-point situation. The remaining pigs (9 swine of both sexes: 8 Duroc × Landrace pigs and 1 male Specipig miniature pig) were exposed to an embolization procedure that uses the LEA Squid-12 (Balt) and the PCT to create an antireflux glue-based plug that traps the detachable part of microcatheter in order to obtain wedge-flow conditions for the Squid-12 distally to the glue-based plug. To do so, the right APA kept the 4F vertebral catheter used for the angiographic study, whereas the left APA was catheterized with a 7F guide catheter (7F ENVOY Catheter, Medos International S.A.R.L.). The experimental design and timeline infographic are depicted in Figure 1B.

Through the 7F guiding catheter, 2 microcatheters were introduced into the left APA: a detachable tip braided microcatheter Sonic 1.2 or 1.5 and a Magic 1.2 or 1.5 microcatheter (Balt Extrusion). A proximal occlusion (embolic plug) with Histoacryl (n-butyl-cyanoacrylate) (B. Braun Surgical S.A.) was performed in the left APA near the left RM wing, trapping the detachable part of the microcatheter and preventing the reflux of the LEA injected through a more distal microcatheter (Figure 2, A and B). The LEA was then injected distally to the embolic plug into the RM until the ICA and CW were filled with the LEA up to the origins of the MCAs. We avoided embolizing the contralateral right wing of the RM to preserve the patency of the contralateral ICA.

Angiographic controls were made through the catheter located in the contralateral APA at different times throughout the procedure. The catheters were removed, the hemostasis of the access point was obtained by manual compression, and the pig was transferred to a 3T MRI scanner.

### Blood sampling.

Blood samples were obtained from anesthetized swine right before the arterial occlusion and at 1 hour, 4 hours, and 1 day after onset of the permanent occlusion. Blood was processed to obtain serum or EDTA plasma, and aliquots were stored at –80°C.

### MRI.

MRI was performed on a Vantage Galan 3T (Canon Medical Systems) using a 16-channel Flex SPEEDER coil, and sequences were adapted from standard clinical protocols. The swine exposed to the occlusion procedure underwent a multiparametric MRI study of the brain at 1.5 hours and again at 1–2 days after producing the occlusion.

The MRI protocol included T1-weighted, T2-weighted, FLAIR, T2 gradient echo (T2*), DTI with 30 directions (baseline image of b = 0 s/mm^2^ and b = 1,000 s/mm^2^), 3D Flow Sensitive Black Blood, 3D Time of Flight, and 3D Dynamic Contrast-Enhanced and T2 Perfusion Dynamic Susceptibility Contrast (DSC) sequences. MRI sequences were acquired in the axial plane using the conditions specified in Supplemental Table 1.

For DSC-perfusion MRI, we used 3.5 mm slices, and image acquisition began 4 seconds after starting injection of a bolus of gadolinium contrast agent (0.1 mL/kg of body weight of Gadovist 1 mmol/mL; Bayer HealthCare Pharmaceutical Inc.) into an ear vein at 5 mL/s with a power injector. This was followed by the injection of 20 mL physiological saline. The data were acquired every 2 seconds for a total of 2 minutes.

### Image postprocessing.

DSC-MRI data were processed using the perfusion software package in Olea Sphere 3.0-SP22 (Olea Medical) for parametric perfusion map computation. Manual arterial pixel selection was chosen for computing an arterial input function. The region within the anterior cerebral artery with the highest contrast enhancement was used as a reference. Cerebral blood volume is defined as the total volume of flowing blood in a given volume of brain and expressed as mL of blood/100 g of brain tissue. Cerebral blood flow refers to the volume of blood moving through a given volume of the brain per unit of time and expressed as mL of blood/100 g of brain tissue/min. In addition, TTP was also calculated, and it corresponds to the time at which contrast concentration reaches its maximum.

Volumetric analyses of MRI sequences were performed with 3D Slicer software. Infarct hyperintense and hemispheric volumes were determined in b = 1,000 DWI sequences within the first 2 days after infarct. Correspondence of hyperintense DWI regions with hypointense regions in ADC maps were verified to avoid possible artifacts or T2 shine-through. Volumetric quantification of the infarct was reviewed by 2 different researchers.

Midline shift analysis was determined in axial T2 images and depicted as the maximal distance between the natural and the cerebral anteroposterior midline T2 images.

### Follow-up and euthanasia.

Long-term analgesia was provided early after the onset of the preneurointerventional procedure using a dermic fentanyl patch (2 μg/kg/h). After both neurointerventional and MRI scanning procedures, the animals were allowed to recover from anesthesia in the preoperating room under mechanical ventilation. Extubation was performed when the animals fully recovered the swallowing reflex and were then returned to their pens to recover overnight under a midazolam regime. During the early postoperative phase, pigs were covered with a thermal foil blanket to avoid heat loss, and later on, pigs had a heater band in the pen floor and a heater lamp located 1.5 meters high and central to the pen during the first night after surgery or neurointervention. Once fully awake, animals had free access to water and food and were video recorded. Neurological deficit was checked (in situ and/or on video recordings) by veterinarians/researchers of the team.

At the end of the follow-up study, swine were euthanized by an i.v. injection of a lethal dose of pentobarbital sodium.

### Histopathological analysis.

Brains were removed and cut into 5 mm coronal sections using a pig brain slicer matrix. Alternate slices of fresh tissue were used for infarct volume assessment by using TTC (Sigma-Aldrich, Chemie Gmbh) to stain live tissue (1.5% TTC at 37°C for 30 minutes in the dark). TTC-unstained tissue was delineated using ImageJ software (NIH) to calculate the overall infarct volume, which is expressed in cm^3^ and as a percentage of the volume of the ipsilateral hemisphere of the brain in each animal. The other slices were fixed in 4% paraformaldehyde at 4°C for 5 days. Upon fixation, each slice was divided into 4 parts and immersed in increasing concentrations of chilled sucrose up to 30%, gently pat dried, embedded in the freezing-embedding compound OCT, frozen in dry ice–chilled isopentane, and stored at –80°C.

Slices (40 μm–thick) from frozen brain fragments were obtained using a cryostat (Leica CM1950) and mounted onto Menzel-Gläser Polysine slides (Thermo Fisher Scientific) that were kept at –20°C. For cresyl violet staining, slides were allowed to thaw at room temperature for 15 minutes, further fixed with methanol at –20°C for 15 minutes, washed in 1× PBS buffer for 2 minutes, and dipped twice in distilled water. Slides were stained in 0.1% cresyl violet (Acros Organics) for 15 minutes, washed twice in distilled water, and dehydrated in increasing concentrations of ethanol. Slides were dried at room temperature for at least 30 minutes and digitalized.

### Determination of biomarkers in serum and plasma.

Quantitative analysis of free amino acids in the pig sera was performed at the Proteomics and Metabolomics core facility at IGTP using the Waters’ Pico Tag method. This method involves precolumn derivatization of the amino acids with phenylisothiocyanate and posterior reverse-phase high-performance liquid chromatography separation adapted from ref. 55. Plasma levels of the molecule HSC70 were determined by Western blot using the mouse anti-HSC70 antibody sc-7298 (Santa Cruz Biotechnology) and the Revert Total Protein Stain (LI-COR) as the loading control.

### Statistics.

Statistical analyses were made on original or log-transformed data using the 2-tailed Student’s *t* test for paired or independent measures, repeated-measures 1-way ANOVA followed by the post-hoc Tukey test, or Pearson correlation, as appropriate. Normal distribution of the data was checked with the Kolmogorov-Smirnov test, or it was checked by the Shapiro-Wilk test when the sample size was too small. When data were not normally distributed and/or there was a serious breach of the homogeneity of variances, nonparametric Mann-Whitney *U* test was used. Statistical analyses were performed using GraphPad Prism v9.3. Results are expressed as the mean and SD. Statistical significance was considered at *P* ≤ 0.05.

### Study approval.

Animal experiments were approved by the Institutional Animal Care and Animal Experimentation Committee of the IGTP and the government of Catalonia (FUE-2019-01138108 and ID 26Q7BHRXN) and were conducted according to international guidelines (EU Directive 2010/63/EU) at the CMCiB following ARRIVE guidelines.

## Author contributions

Conceptualization was contributed by AD, CC, and TG. Investigation and laboratory work were contributed by CC, MMS, AGS, NDR, OP, and MRGS. Data curation and statistics were contributed by OMS, MHP, JP, AVC, AIS, and APG. Original draft preparation and writing, review, and editing of the manuscript were contributed by MMS, CC, OMS, and TG. Project administration was contributed by JG and TG. Funding acquisition was contributed by AD and TG. All authors have read and agreed to the published version of the manuscript. The 2 first coauthors have made equal contributions to the study and share merit and position; the final method for author ordering was determined alphabetically.

## Supplementary Material

Supplemental data

Supplemental video 2

Supplemental video 3

## Version 1 02/28/2023

In-Press Preview

## Version 2 04/24/2023

Electronic publication
