# Supplementary material for: Establishment of a reproducible and minimally invasive ischemic stroke model in swine
Source: JCI Insight. 2023 Apr 24;8(8):e163398. doi: 10.1172/jci.insight.163398 (PMC10243803; doi:10.1172/jci.insight.163398)
Supplement: Supplemental data [file jciinsight-8-163398-s152.pdf]

Supplementary Materials for

ESTABLISHMENT OF A REPRODUCIBLE AND MINIMALLY INVASIVE ISCHEMIC STROKE MODEL IN SWINE

Carlos Castaño *et al*.

· Videos 1, 2 and 3 are submitted separately to illustrate post-stroke pig's behavior.

We provide three videos of the pig's performance post-stroke onset, while left undisturbed in its pen. Each video shows several minutes of the pig's behavior at x15 speed and, hence, in less than 40 seconds. Of note, increased speed of the video might mislead the viewer to think that the pig is falling when, in fact, the pig is normally lying down.

· This file includes:

Figures S1 to S5

Tables S1 to S3

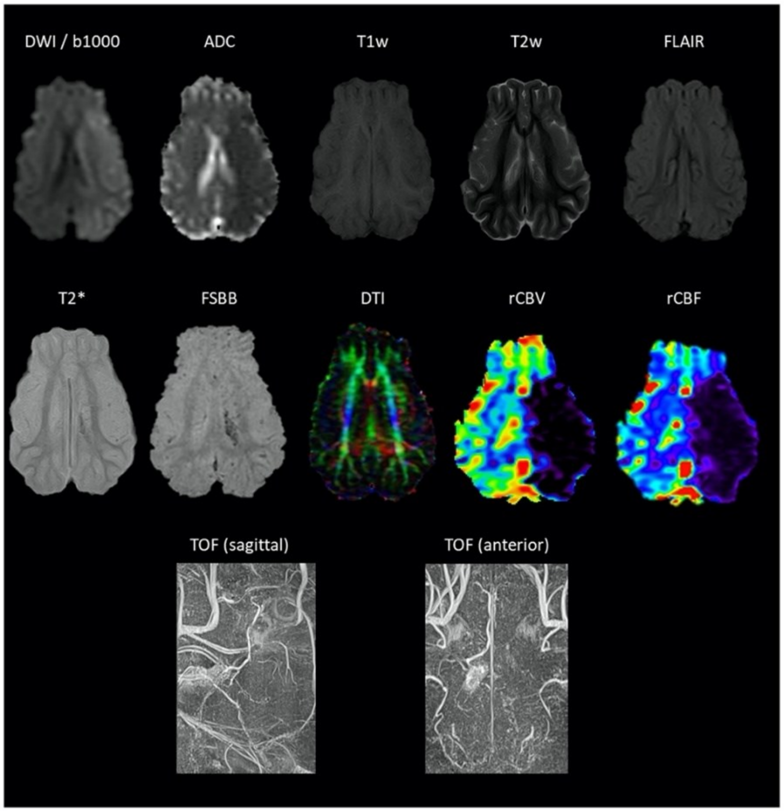

Figure S1. Representative images obtained 90 minutes after the ischemia onset for diffusion weighted imaging (DWI)/b1000), apparent diffusion coefficient (ADC), T1-weighted (T1w), T2-weighted (T2w), Fluid Attenuated Inversion Recovery (FLAIR), T2 gradient echo (T2*), 3D Flow Sensitive Black Blood (FSBB), Diffusion Tensor Imaging (DTI), 3D Dynamic Contrast Enhanced (DCE) and T2 Perfusion Dynamic Susceptibility Contrast (DSC). DSC-MRI data was processed using the perfusion software package in Olea Sphere 3.0-SP22 to generate rCBV and rCBF images. Representative images of 3D Time of Flight (TOF) sequences in sagital and anterior view were depicted. Note that the lack of contrast in the left hemisphere in TOF (anterior) image corresponds with the hemisphere showing large areas of compromised blood flow in rCBV and rCBF. Left hemispheric areas show early signs of ischemic damage in the DWI and ADC images.

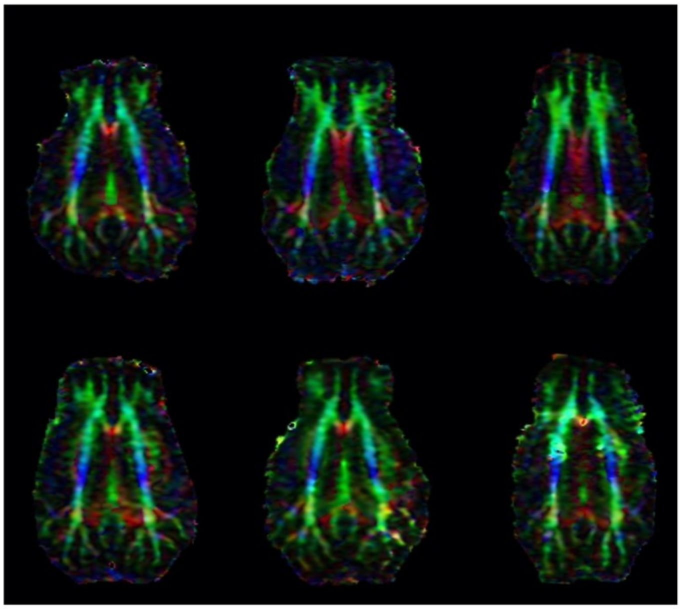

Figure S2. Diffusion Tensor Imaging of 6 different pigs 90 minutes after the successful embolization of the RM+left CW wing. Color codes of the tract orientation are as follows: red for lateral axis fibers, green for anteroposterior axis fibers, and blue for dorsoventral axis fibers. In each brain image, the white matter tracts present in the left and the right hemisphere are similar.

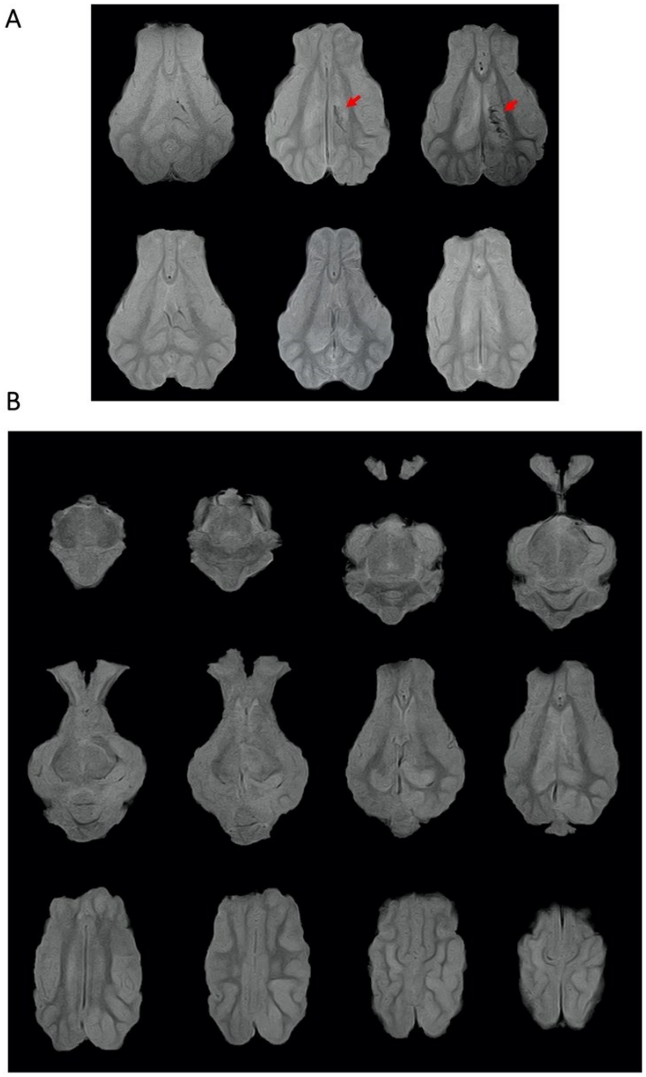

Figure S3. **(A)** T2 gradient echo (T2*) of 6 different pigs 90 minutes after the successful embolization of the RM+left CW wing. No typical signs of hemorrhage were observed in T2* gradient echo, but T2* sequence depicts in some of the brains (see red arrows) subcortical hypointensities contained in specific brain structures that will be further investigated. **(B)** Representative superior-inferior collection of T2 gradient echo T2* images obtained 1 day after the permanent embolization of the RM+left CW wing in one of the pigs.

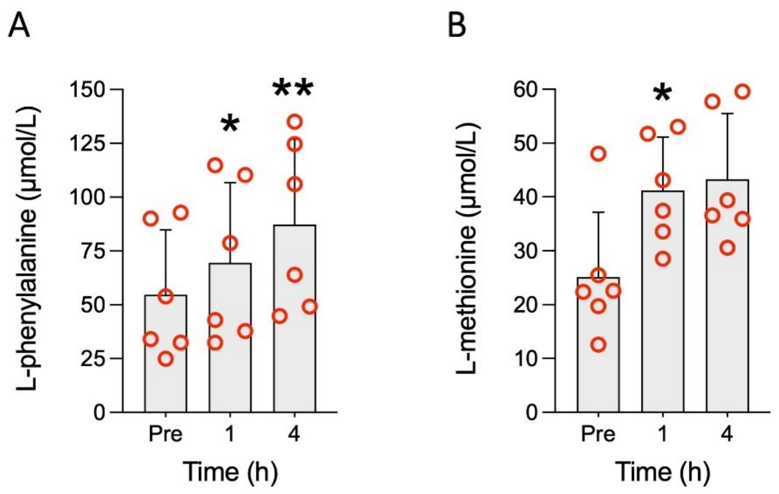

Figure S4. Time-course effect of stroke on the total serum levels of **(A)** L-phenylalanine and **(B)** L-methionine previously (Pre) and 1 and 4 hours after the onset of stroke. Of note, phenylalanine has been found increased in most clinical trials of ischemic stroke and increased levels of L-methionine have been recently reported in young stroke patients (https://doi.org/10.1007/s11306-021-01774-7). Our experimental stroke model was performed in young Duroc x Landrace swine and in adult miniature pigs. * $p<0.05$, ** $p<0.01$ vs Pre. Mean and SD are shown.

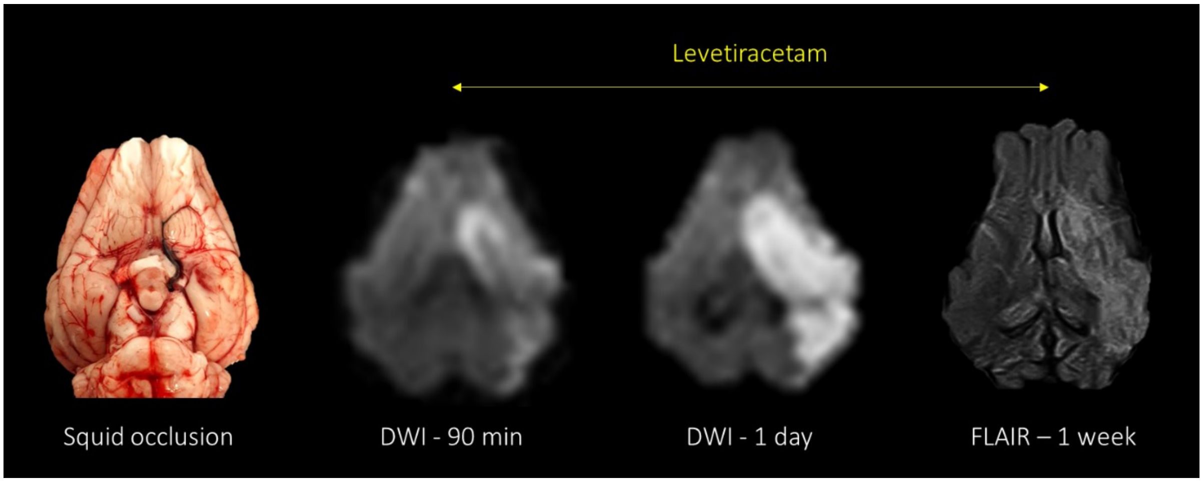

Figure S5. Representative images of post-mortem assessment of the squid location (left), and of the longitudinal infarct evolution (using DWI or FLAIR MRI sequences) in pigs that have been daily medicated with the antiepileptic levetiracetam after the stroke onset. The infarct is observed as the white/hyperintense area in the pig brain MRI images obtained 90 min, 1 day, and 1 week after the induction of stroke. As Supplementary Material we provide videos of the pig's performance 1 day (video 2) and 1 week (video 3) post-stroke onset, while left undisturbed in its pen; images in this Figure and videos were taken from the same pig. Each video shows several min of the pig's behavior at x15 speed and, hence, in less than 40 seconds.

	FOV (mm)	Matrix (mm)	TR (ms)	TE (ms)	TI (ms)	Flip angle	Slice thickness (mm)	In-plane resolution (mm)	Scanning time (min)
**T1-weighted**	100x171	176x304	700	9	-	70	3	0.28x0.28	7:07
**T2-weighted**	100x170	224x384	5300	105	-	90	3	0.22x0.22	6:49
**FLAIR**	100x170	128x192	8000	120	2200	90	3	0.39x0.39	7:44
**T2 gradient echo (T2*)**	100x170	240x256	600	12	-	20	3	0.21x0.21	5:32
**DTI with 30 directions**	170x105	80x168	6799	90	-	90	2	0.51x0.51	11:14
**3D FSBB (Flow Sensitive Black Blood)**	100x169	144x240	39	30	-	10	1	0.35x0.35	6:52
**3D TOF**	155x155	256x256	23	3.9	-	15	0.6	0.30x0.30	8:00
**3D T1 Dynamic (DCE)**	146x235	144x128	6.9	0.9	-	18	10	0.51x0.51	2:22
**T2 Perfusion (DSC)**	157x236	96x128	2000	25	-	90	3.5	0.61x0.61	2:00

Table S1. Table depicting the conditions used for MRI axial plane sequence acquisition on a Vantage Galan 3T (Canon Medical Systems, Tochigi, Japan) using a 16 channel Flex SPEEDER coil.

A

Structure	Cortex	Thalamus	Striatum	Limbic	Epithalamus	Hypothalamus
% Pigs affected at 90 min	100	83	83	100	50	100
% Pigs affected at 1-2 d	100	83	100	100	67	100

B

Structure	Area	% Pigs affected at	
		90 min	1-2 d
Cerebral cortex	Primary somatosensory cortex	67	83
	Primary motor cortex	0	0
	Somatosensory association cortex	83	100
	Dorsolateral prefrontal cortex	17	17
	Anterior prefrontal cortex	0	33
	Orbitofrontal cortex	17	50
	Insular cortex	83	100
	Primary visual cortex (V1)	100	100
	Secondary visual cortex (V2)	100	100
	Associative visual cortex (V3)	100	100
	Inferior temporal gyrus	100	100
	Middle temporal gyrus	100	100
	Superior temporal gyrus	100	100
	Dorsal anterior cingulate cortex	33	50
	Anterior entorhinal cortex	83	83
	Parahippocampal cortex	100	100
	Fusiform gyrus	100	100
	Prepiriform area	100	100
	Premotor cortex*	50	33
	Ventral posterior cingulate cortex*	83	83
	Ventral anterior cingulate cortex*	33	67
	Ectosplenial area*	50	67
	Piriform cortex*	100	100
	Retrosplenial cingular cortex*	50	50
	Dorsal posterior cingular cortex*	83	83
	Perirhinal cortex*	83	100
	Auditory cortex*	100	100
	Olfactory bulb*	nd	nd

Structure	Nucleus	% Pigs affected at	
		90 min	1-2 d
Thalamus	Pulvinar nuclei	67	67
	Reticular thalamic nucleus	83	83
	Ventral anterior thalamic nucleus	83	83
	Ventral posterior thalamic nucleus	83	83
	Anteroventral thalamic nucleus*	83	67
	Central thalamic area*	67	83
	Laterodorsal thalamic nucleus*	67	67
	Geniculate nuclei*	67	83
	Mediodorsal thalamic nucleus*	83	83
Striatum	Caudate nucleus	67	83
	Claustrum	83	100
	Globus pallidus	83	83
	Putamen	83	100
	Accumbens nucleus*	50	50
	Substantia nigra*	50	83
Limbic system	Fornix	67	100
	Hippocampus	83	100
	Subiculum	67	67
	Amygdala	100	100
Subthalamus	Subthalamic nucleus	67	83
	Zona incerta	67	83
Epithalamus	Habenular nuclei	50	67
	Pineal gland	nd	nd
Hypothalamus	Anterior hypothalamic area	100	100
	Mamillary body	50	67
	Medial hypothalamic area	83	100

* Areas/nuclei not shown in Scheulin et al., Sci.Rep. 2021; 11:3814.

Table S2. Percentage of pigs exposed to the ischemic stroke model showing ischemic lesions at 90 min and 1-2 days in **(A)** large brain structures and **(B)** in specific areas/nuclei within. Lesion was measured in each brain structure/area/nucleus 90 min after the embolization, when the blood flow through the MCAs has already been prevented, and it was measured again 1-2 days later, when tissue has been exposed to permanent occlusion for more than 1 day; nd: not determined.

	Time before occlusion			Occlusion	Time post-occlusion		
Temperature (ºC)	60 min	30 min	10 min	0 min	10 min	30 min	60 min
Pig 1	37.5	37.2	37.2	na	37.1	na	na
Pig 2	38.0	37.8	37.2	na	na	36.9	36.5
Pig 3	38.3	38.1	38.0	na	37.9	37.9	na
Pig 4	37.9	37.6	37.1	na	37.0	na	na
Pig 5	37.2	36.8	36.8	36.6	36.6	37.2	37.0
Pig 6	37.0	37.3	37.5	38.0	38.2	38.4	na
Heart rate (bpm)	60 min	30 min	10 min	0 min	10 min	30 min	60 min
Pig 1	165	155	155	na	140	na	na
Pig 2	95	97	94	na	na	93	83
Pig 3	85	85	104	na	90	na	94
Pig 4	83	82	80	na	70	73	68
Pig 5	125	100	92	90	90	88	91
Pig 6	142	112	96	88	83	81	81
SpO_2 (%)	60 min	30 min	10 min	0 min	10 min	30 min	60 min
Pig 1	100	100	98	na	100	na	na
Pig 2	100	100	100	na	na	100	98
Pig 3	97	98	99	na	100	na	98
Pig 4	95	96	96	na	98	95	98
Pig 5	100	100	100	100	100	100	100
Pig 6	97	97	98	97	97	97	100
CO_2 (mmHg)	60 min	30 min	10 min	0 min	10 min	30 min	60 min
Pig 1	37	33	35	na	35	na	na
Pig 2	37	37	36	na	na	37	35
Pig 3	36	39	40	na	38	na	29
Pig 4	33	35	33	na	36	35	35
Pig 5	46	48	46	48	49	47	40
Pig 6	45	40	39	38	35	38	38
MABP (mmHg)	60 min	30 min	10 min	0 min	10 min	30 min	60 min
Pig 1	60 - 70	55 - 65	55 - 65	na	75 - 85	na	na
Pig 2	55 - 65	70 - 80	75 - 85	na	na	70 - 80	45 - 55
Pig 3	75 - 85	100 - 110	70 - 80	na	85 - 95	na	70 - 80
Pig 4	65 - 75	60 - 70	55 - 65	na	65 - 75	55 - 65	45 - 55
Pig 5	70 - 80	70 - 80	90 - 100	90 - 100	70 - 80	45 - 55	65 - 75
Pig 6	105 - 115	65 - 75	70 - 80	85 - 95	65 - 75	105 - 115	45 - 55

na: not available.

Table S3. Values of temperature, heart rate, %SpO_2, CO_2 and mean arterial blood pressure (MABP) 60, 30 and 10 minutes before the effective MCAs occlusion, while the pig was exposed to the neurointerventionism procedures, at the time of MCAs occlusion (0 min in the Table), and 10, 30 and 60 minutes after the occlusion onset.
